# Remote coaching with blood flow restriction training: impact on strength, body composition, metabolism, and walking energetics in overweight adults

**DOI:** 10.1007/s00421-026-06422-3

**Published:** 2026-09-08

**Authors:** Tom Citherlet, Sébastien Christe, Améline Cattin, Gustavo R. Mota, Grégoire P. Millet

**Affiliations:** 1 Institute of Sport Sciences, University of Lausanne, Synathlon, office 3224, 1015 Lausanne, Switzerland; 2 Institute of Health Sciences, Federal University of Triângulo Mineiro, Uberaba, Brazil

**Keywords:** KAATSU, Resistance training, Obesity, Fat mass, Lean mass, Gait

## Abstract

**Purpose:**

Blood flow restriction (BFR) is a widely used exercise modality in sport; however, evidence supporting its use in individuals with overweight remains limited. This study evaluated whether home-based BFR training provides additional benefits over conventional resistance training in this population.

**Methods:**

Twenty-seven participants were assigned to BFR (*n* = 13) or non-BFR (*n* = 14) groups for a 6-week (3x/week) home-based strength program via videoconference. Assessments included body composition (DEXA), glucose and lipid metabolism, knee extensor/flexor strength, and gait analysis (energetics, biomechanics and preferred walking speed).

**Results:**

No group × time interactions were observed for any outcome, indicating no BFR-induced benefits. Across both groups, trunk lean mass increased (BFR: 27.2 ± 4.3 to 27.9 ± 4.5 kg; non-BFR: 25.4 ± 4.0 to 25.8 ± 4.4 kg; *P* = 0.007). HDL-cholesterol decreased (*P* = 0.008) and glycated haemoglobin increased (*P* < 0.001), both statistically significant but clinically negligible and directionally unfavourable. Isometric knee-extension peak torque increased (BFR: 186 ± 50 to 222 ± 80 Nm; non-BFR: 183 ± 81 to 204 ± 90 Nm; *P* < 0.001), with parallel gains in isokinetic concentric extension and flexion at 30°/s, 90°/s, and 180°/s (all *P* ≤ 0.032). Gait analysis showed no changes.

**Conclusion:**

Six weeks of remotely supervised bodyweight resistance training improved knee extensor and flexor strength in sedentary adults with overweight or obesity. Under the present conditions, adding BFR did not provide evidence of additional benefit for strength, body composition, metabolic markers, or walking outcomes.

**Trial registration:**

The trial was registered in advance at ClinicalTrials.gov with the identifier NCT05371119 on 12 May 2022.

## Introduction

Societal advancements have increased caloric availability and reduced physical demands (Church and Martin 2018), reversing historical challenges. Obesity now kills more than malnutrition, with global prevalence of obesity/overweight projected to reach 51% by 2035 (World Obesity Federation 2023). Simultaneously, ~ 70% of the population is sedentary, and 27% of adults fail to meet recommended activity levels (World Health Organization 2002, 2022). These factors are primary drivers of non-communicable diseases (Peters et al. 2019) and impose major economic burdens: physical inactivity costs global healthcare INT$53.8 billion yearly (Ding et al. 2016), while obesity takes  ≈ 21% of US healthcare spending (Cawley and Meyerhoefer 2012). Therefore, developing effective interventions is essential to address these public health challenges.

Blood flow restriction (BFR) has emerged as an effective training modality across diverse populations, including athletes, injured individuals (Hughes et al. 2017), older adults (Centner et al. 2019), and also individuals with overweight and obesity (Kong et al. 2025). This training modality involves applying cuffs to the proximal portions of the limbs. By partially restricting blood flow to the working muscles during exercise, BFR training induces unique physiological adaptations through metabolic stress, muscle fiber recruitment changes, and cell swelling (Guedria et al. 2022), offering individuals with obesity a low-impact method to significantly improve muscle strength and hypertrophy (Perera et al. 2022), cardiovascular fitness (Formiga et al. 2020), body fat (Sun 2022), lipid profiles (Sun 2022), and glucose tolerance (Saatmann et al. 2021). Energetics and biomechanics of walking, such as net energy cost of walking and preferred walking speed (Fernández Menéndez et al. 2019), deteriorate with obesity but can improve with both endurance and strength training (Van Abbema et al. 2015), although these alterations are not uniform across all obesity classes (Primavesi et al. 2021; Tucker et al. 2024); however, it is not known whether BFR can further enhance these improvements. In addition, the higher cardiovascular demands (Silva et al. 2019), increased energy expenditure (Karabulut and Garcia 2017), and the decreased number of repetitions to attain failure with BFR (Gavanda et al. 2020) could lead to similar benefits in a shorter time (Karabulut and Garcia 2017), which can be an additional reason to engage in BFR training for individuals with obesity, leading to greater adherence to the program (Allison et al. 1999).

Furthermore, while BFR has emerged as a promising training modality across diverse populations, the existing evidence base is skewed toward younger, active cohorts or older adults with age-related decline, leaving a notable gap in middle-aged adults (e.g., 30–60 y). This demographic is particularly vulnerable to the accumulation of sedentary behaviour and rising obesity prevalence, yet the feasibility and efficacy of remotely delivered, low-load BFR training have not been rigorously tested in this population.

Home-based training program (e.g., conducted by videoconference) presents a compelling avenue to enhance sports participation and adherence among various populations, including individuals with obesity. It addresses barriers faced by these individuals, such as discomfort exercising in public (Thedinga et al. 2021), transportation limitations, and global shutdowns (e.g., the COVID-19 pandemic). Consequently, digital home-based infrastructure provides a resilient framework for maintaining physical activity when traditional facilities are inaccessible.

This study evaluated whether a home-based blood flow restriction (BFR) resistance training program provides superior benefits compared to non-BFR training in individuals with overweight or obesity. We specifically assessed outcomes in glucose and lipid metabolism, knee flexor and extensor strength, and gait energetics/biomechanics. We hypothesized that BFR training would yield greater improvements than traditional resistance training across four key domains: (1) body composition, characterized by increased lean mass and decreased fat mass; (2) metabolic health; (3) muscular strength; and (4) walking biomechanical function.

## Methods

### Ethics approval

This clinical trial was approved by the local ethics committee (CER-VD: 2021-01550), registered at ClinicalTrials.gov (NCT05371119), and adhered to the most recent guidelines outlined in the Declaration of Helsinki. Prior to inclusion, all participants provided written informed consent.

### Participants

Thirty sedentary adults with overweight and obesity were recruited in this study but three dropped out: two for health reasons (confirmed medically as not related to the protocol) and one due to absence at post-tests. Participants were pair-matched by body mass index (BMI) for practical stratified randomization at recruitment and then randomly assigned to blood flow restriction (BFR; *n* = 13, 8 men, 5 women, age = 46 ± 12 year, height = 1.73 ± 0.10 m, body mass = 89.1 ± 8.9 kg, BMI = 30 ± 4 kg/m^2^) and active control (non-BFR; *n* = 14, 4 men, 10 women, age = 44 ± 10 year, height = 1.66 ± 0.10 m, body mass = 83.5 ± 12.4 kg, BMI = 30 ± 5 kg/m^2^) groups.

Participants were required to be between 30 and 60 years old, with a BMI ranging from 25 to 35 kg/m^2^ and have a physical activity  ≤ 150 min per week. They were also required to be free from high blood pressure (≤ 140/90 mmHg), cardiovascular disease, or severe type 2 diabetes and not be undergoing medical treatment for lipid profile, blood glucose, or blood pressure.

### Experimental protocol

**Fig. 1:**
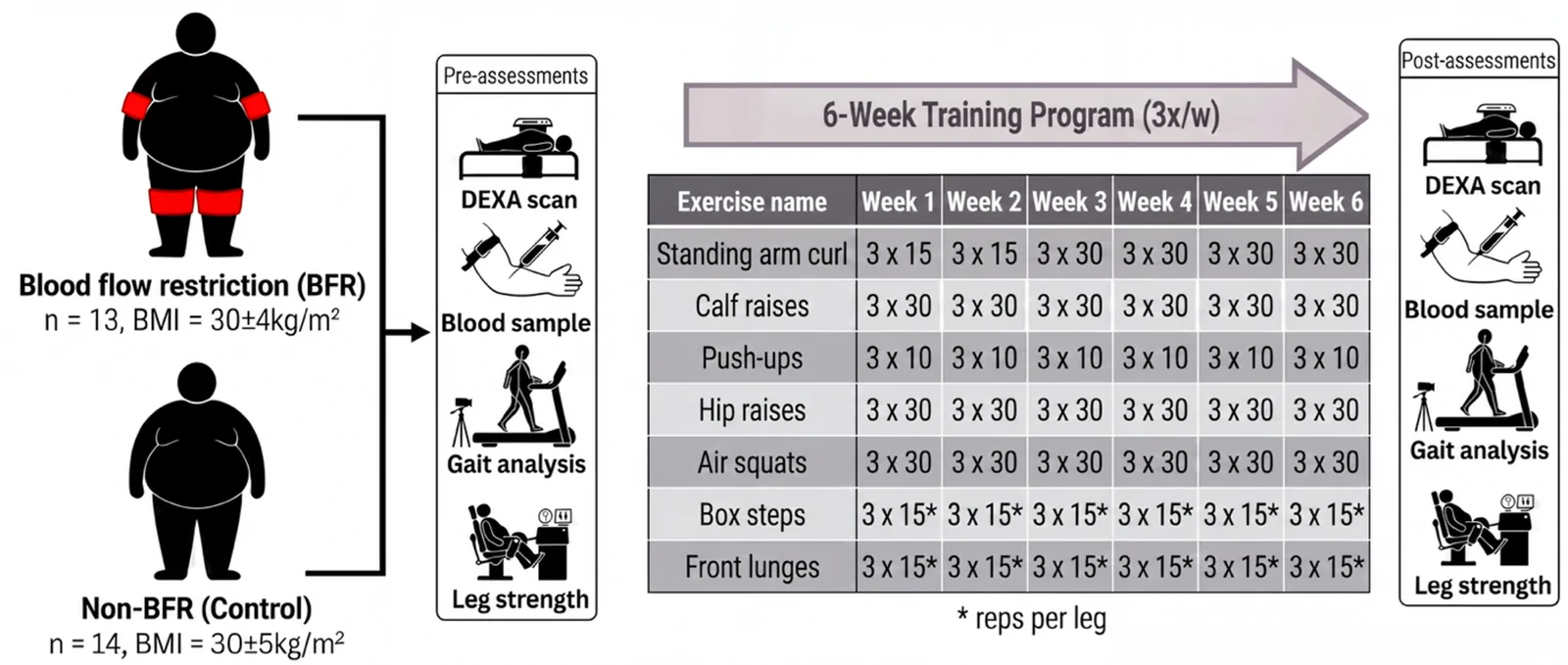
Study design. Participants were pair-matched by BMI to either blood flow restriction (BFR) or active control (non-BFR) and completed the same 6-week home-based program, with identical pre- and post-assessments. Red bands indicate the pneumatic cuffs worn by the BFR group throughout every training session.

Participants took part in a 6-week training program with assessments conducted before and after, within 2 weeks (Fig. 1).

### Training

Participants completed a 6-week bodyweight strength training program at home under the guidance and supervision of a qualified trainer using the Zoom application (Zoom Video Communications Inc., San Jose, CA, USA) due to the COVID-19 pandemic. Training occurred three times weekly during the six weeks (18 sessions in total), as neuromuscular changes are detectable within 4–6 weeks. Each 30–35 min session included seven exercises (standing arm curl, calf raises, push-ups, hip raises, air squats, box steps, and front lunges) for 3 sets of 10–30 reps, performed at a slow tempo, with rest periods reduced from 60 to 30 s over time to progressively increase training density.

Participants in the BFR group trained with elastic pneumatic cuffs (BStrong, Park City, UT, USA) provided during pre-tests, sized according to limb circumference. During the pre-test visit, participants were instructed and practiced correct cuff placement and inflation procedures. Cuffs were kept inflated throughout the entire session at an absolute pressure of 250 mmHg on the arms and 400 mmHg on the legs, based on our prior validation work in the same elastic cuff system demonstrating meaningful reductions in limb blood flow at these pressures (Citherlet et al. 2022). However, during the initial session, lower pressures of 150 mmHg for the arms and 250 mmHg for the legs were applied to induce gradual adaptation.

Adherence was recorded for every session. Because sessions were delivered live by videoconference, the trainer confirmed attendance and completion in real time and, for the BFR group, could provide immediate assistance and corrective guidance if cuff positioning or inflation required adjustment. All retained participants completed the 18 prescribed sessions. No adverse events (e.g., syncope, venous thromboembolic event, persistent numbness, or skin injury) were reported.

### Testing

Each assessment comprised body composition (DEXA), blood sampling, knee flexor and extensor strength, and treadmill walking biomechanics distributed over several days according to individual scheduling.

Body composition was measured by dual-energy X-ray absorptiometry (DEXA, Hologic Inc, Bedford, MA, USA) and quantified total, fat, and lean body mass. Segmental arm and leg lean mass are reported as the mean of the left and right limb.

Blood samples were collected by an accredited nurse to assess carbohydrate and lipid metabolism. The participants were required to observe a fasting period on the day preceding the blood sampling (no food or sweet drinks after midnight). Venous blood samples were collected from the antecubital vein after overnight fasting and analyzed in a certified laboratory using standardized enzymatic methods. Lipid metabolism was evaluated through measurements of cholesterol levels, triglycerides, HDL cholesterol, and the total cholesterol to HDL ratio. Carbohydrate metabolism was assessed by measuring fasting blood glucose levels, along with glycated haemoglobin (HbA1c).

Muscular strength of the dominant leg was measured. Knee extensors and flexors were measured using a dynamometer (ConTrex MJ, CMV AG, Dubendorf, Switzerland). Belts were firmly secured to the tested thigh and the trunk to minimize compensatory movements. Every test began with a submaximal familiarization phase, which was then followed by the main testing phase. Initially, isometric knee extensor strength was evaluated by recording the highest peak torque generated over three repetitions at a 90° angle during a maximal 5-s effort. Subsequently, isokinetic concentric knee extensor and flexor strength were assessed at angular velocities of 30°/s, 90°/s, and 180°/s by recording the best peak torque generated over 3 repetitions. Strong verbal encouragements were provided throughout, and visual feedback was withheld. The dynamometer chair settings were established during the initial visit for each participant and replicated during the subsequent visit to ensure measurement reproducibility.

Gait energetics, preferred walking speed, and gait biomechanics were evaluated. Gait energetic measurements consisted of quantifying the energy cost of treadmill walking. Gas exchanges were measured breath-by-breath with a calibrated ergospirometer (Quark CPET, Cosmed, Rome, Italy) in a standing position for 5 min (standing metabolic rate) and while walking at 1.11 m/s for 6 min on a treadmill (T10-FMT-MED, Arsalis, Belgium, Louvain-la-Neuve). A fixed speed of 1.11 m/s (4 km/h) was used to standardize gait energetics and mechanics across assessments and participants (Fernández Menéndez et al. 2019, 2020; Malatesta et al. 2022). The standing and walking gross metabolic rates were evaluated based on steady-state measurements of oxygen uptake (${\dot{\text {VO}}_{2}}$) and carbon dioxide production (${\dot{\text {VCO}}_{2}}$) using Brockway’s standard equation (Brockway 1987). The respiratory exchange ratio values remained  < 1.0 throughout all trials, indicating that energy was primarily derived from oxidative metabolism. The net metabolic rate was calculated by subtracting the standing metabolic rate from the gross walking metabolic rate. To calculate the gross and net energy cost of walking, the gross and net metabolic rates were divided by the walking speed, respectively (di Prampero 1986). Absolute and net energy cost of walking values (J/m) were then normalized by body mass (J/kg/m). Standing metabolic rate was used to calculate the net energy cost of walking because it provides a more accurate estimation of the energy cost of walking by disregarding the metabolic cost associated with supporting body weight and maintaining balance during walking, particularly in participants with overweight or obesity (Peyrot et al. 2010).

The overground preferred walking speed was established using an adapted version of a protocol previously described (Dal et al. 2010). Participants performed three walking trials at their “most comfortable pace” along a 20-m walkway. Infrared timing gates (WittyGate, Microgate, Bolzano, Italy), positioned at 5 m and 15 m, measured the time taken to cover the central 10-m stretch. The preferred walking speed was calculated for each trial, and the average of two best trials was used for analysis. During these trials, the preferred walking speed was recorded using an inertial sensor system (Physilog^®^, GaitUp, Lausanne, Switzerland).

The biomechanics of treadmill walking (spatiotemporal parameters, external work, and pendular recovery) were assessed with an instrumented treadmill as previously described (Fernández Menéndez et al. 2020). Briefly, 10 strides from the last 30 s of the 6-min walk bout were selected to obtain the vertical, forward, and lateral ground reaction forces. The latter were used in conjunction with the participant’s mass to calculate the 3D accelerations of the center of mass. The mathematical integration of these 3D accelerations provided the velocity changes of the center of mass along the vertical (*V*_*v*_), horizontal (*V*_*h*_), and lateral (*V*_*l*_) axes.

The total kinetic energy (*Ek*) was obtained by summing the vertical (*Ekv*), horizontal (*Ekh*), and lateral (*Ekl*) kinetic energy using the body mass (*m*) and the velocity changes (Eq. 1).

The potential energy (*Ep*) was computed with the vertical position of the center of mass (*h*; obtained by mathematical integration of *Vv*), gravity acceleration (*g* = 9.81 m/s^2^), and *m* (Eq. 2).

The total mechanical energy (*Etot*) was determined by summing the increments in *E*_*k*_ and *E*_*p*_ (Eq. 3).

1 $$\:{\mathrm{E}}_{\mathrm{k}}={\mathrm{E}}_{\mathrm{kv}}+{\mathrm{E}}_{\mathrm{kh}}+{\mathrm{E}}_{\mathrm{kl}}=0.5\:\mathrm{m}\:\left({\mathrm{V}}_{{\mathrm{v}}^{2}}+{\mathrm{V}}_{{\mathrm{h}}^{2}}+{\mathrm{V}}_{{\mathrm{l}}^{2}}\right)$$

2 $$\:{\mathrm{E}}_{\mathrm{p}}=\mathrm{m}\times\:\mathrm{g}\times\:\mathrm{h}$$

3 $$\:{\mathrm{E}}_{\mathrm{tot}}={\mathrm{E}}_{\mathrm{k}}+{\mathrm{E}}_{\mathrm{p}}$$

External mechanical work (Wext) (J kg⁻¹ m⁻¹) was then calculated from the sum of the positive increments of the Etot curve of each step and then divided by step length (Massaad et al. 2007; Cavagna et al. 1963, 1976). An average of the values obtained from the 20 steps was then calculated.

*Recovery* describes the fraction of mechanical energy recovered with pendular-like mechanism. It was calculated with the amount of external mechanical work performed per step (W_ext_), defined as the sum of the positive increments in *E*_*tot*_, as well as the sum of the increments and decrements in the *E*_*k*_ curves (${\mathrm{W}}_{\mathrm{k}}$ and $\:{\mathrm{W}}_{\mathrm{k}-}$, respectively), and the sum of the increments and decrements in the *E*_*p*_ curves (${\mathrm{W}}_{\mathrm{p}}$ and $\:{\mathrm{W}}_{\mathrm{p}-}$, respectively) (Malatesta et al. 2022) (Eq. 4).

4 $$\begin{aligned} \mathrm{Recovery}\:(\%) & = \frac{{\mathrm{W}}_{\mathrm{k}}+{\mathrm{W}}_{\mathrm{p}}+\:{\mathrm{W}}_{\mathrm{k}-}+{\mathrm{W}}_{\mathrm{p}-\:}-{\mathrm{W}}_{\mathrm{ext}-}\:-{\mathrm{W}}_{\mathrm{ext}}}{{\mathrm{W}}_{\mathrm{k}}+{\mathrm{W}}_{\mathrm{p}}+\:{\mathrm{W}}_{\mathrm{k}-}+{\mathrm{W}}_{\mathrm{p}-}} \times\:100 \end{aligned}$$

The net locomotor efficiency was calculated as the ratio of *W*_*ext*_ to the net energy cost of walking (Fernández Menéndez et al. 2018). 

### Data and statistical analysis

A priori power analysis for a repeated-measures ANOVA (group × time interaction) was conducted using G*Power 3.1.9.6. The calculation was based on the primary outcome, the training-induced gain in lean mass (f = 0.39; Loenneke et al. 2012), indicated that 20 participants were required (α = 0.05, power = 0.90). Muscle strength and lean mass were the primary outcomes; body composition sub-measures, glucose and lipid markers, and gait outcomes were secondary (i.e. the study was not powered to detect differences in these variables). The analysis assumed 2 groups, 2 measurements, a repeated-measures correlation of 0.5, and ε = 1.

A two-way ANOVA was conducted to assess the effects of training (Pre vs. Post tests), group (Non-BFR vs. BFR), and their interaction. In case of missing value, a mixed-effect model was utilized. Subsequently, post-hoc comparisons between group means were performed using the Tukey multiple comparison test. For every outcome, exact P values are reported together with effect sizes (partial eta-squared) and, for within-group pre-to-post changes, Cohen’s dz with 95% confidence intervals. All statistical analyses were carried out using GraphPad Prism software (version 9.5.0, San Diego, CA), and statistical significance was set at *P* ≤ 0.05. As a sensitivity analysis, the group × time comparisons for the primary and key secondary outcomes were repeated with sex included as a covariate; no interaction became significant, indicating that the between-group findings are robust to the baseline sex imbalance. Given the number of exploratory outcomes, isolated significant time effects of small magnitude are interpreted cautiously as hypothesis-generating rather than confirmatory.

## Results

Table 1 presents body composition results. Blood markers are displayed in Table 2, strength results in Table 3, and gait biomechanic/energetics measurements in Table 4.

**Table 1:** Body composition

Groups	BFR		Non-BFR		Time effect	Group effect	Interaction effect	BFR		Non-BFR	
	Pre	Post	Pre	Post				dz	95% CI	dz	95% CI
Total body mass (kg)	89.1 ± 8.9	89.2 ± 9.2	83.5 ± 12.4	82.4 ± 12.2	0.181 (0.08)	0.151 (0.08)	0.109 (0.10)	+ 0.05	(− 1.1, 1.3)	− 0.64	(− 2.0, − 0.1)
Body mass index (kg/m^2^)	29.9 ± 4.3	30.0 ± 4.5	30.4 ± 4.7	30.0 ± 4.4	0.154 (0.09)	0.898 (0.00)	0.088 (0.11)	+ 0.06	(− 0.4, 0.4)	− 0.67	(− 0.7, − 0.1)
Total fat (%)	35.6 ± 8.1	35.2 ± 8.3	37.1 ± 7.5	36.8 ± 8.3	0.139 (0.08)	0.622 (0.01)	0.831 (0.00)	− 0.52	(− 0.9, 0.1)	− 0.20	(− 1.2, 0.6)
Arms fat (%)	36.8 ± 11.7	36.2 ± 11.6	40.6 ± 10.8	40.8 ± 11.1	0.609 (0.01)	0.345 (0.04)	0.292 (0.04)	− 0.34	(− 1.6, 0.5)	+ 0.10	(− 1.0, 1.4)
Trunk fat (%)	36.6 ± 6.8	36.2 ± 7.3	36.3 ± 6.0	36.1 ± 7.2	0.303 (0.04)	0.930 (0.00)	0.810 (0.00)	− 0.45	(− 0.9, 0.1)	− 0.12	(− 1.4, 0.9)
Leg fat (%)	34.9 ± 11.2	34.6 ± 11.3	39.1 ± 10.4	38.6 ± 11.0	0.097 (0.11)	0.345 (0.04)	0.645 (0.01)	− 0.32	(− 0.9, 0.3)	− 0.36	(− 1.4, 0.3)
Visceral fat (g)	882 ± 314	889 ± 300	772 ± 255	777 ± 225	0.750 (0.00)	0.297 (0.04)	0.944 (0.00)	+ 0.11	(− 34, 49)	+ 0.04	(− 68, 78)
Total lean mass (kg)	54.6 ± 8.9	55.6 ± 9.1	50.2 ± 8.9	50.0 ± 9.3	0.147 (0.08)	0.163 (0.08)	0.055 (0.14)	+ 0.70	(0.1, 1.9)	− 0.10	(− 1.0, 0.7)
Arm lean mass, per limb (kg)	3.2 ± 0.9	3.2 ± 1.0	2.8 ± 0.9	2.6 ± 0.7	0.120 (0.10)	0.137 (0.09)	0.247 (0.06)	− 0.06	(− 0.24, 0.20)	− 0.60	(− 0.32, − 0.01)
Trunk lean mass (kg)	27.2 ± 4.3	27.9 ± 4.5	25.4 ± 4.0	25.8 ± 4.4	**0.007** (0.25)	0.242 (0.05)	0.407 (0.03)	+ 0.71	(0.1, 1.4)	+ 0.41	(− 0.2, 1.0)
Leg lean mass, per limb (kg)	9.2 ± 1.6	9.1 ± 1.5	8.2 ± 1.8	8.0 ± 1.9	0.178 (0.07)	0.127 (0.09)	0.492 (0.02)	− 0.14	(− 0.29, 0.18)	− 0.38	(− 0.43, 0.09)
Total fat/size^2^ ratio (kg/m^2^)	10.3 ± 3.6	10.3 ± 3.7	11.0 ± 3.6	10.8 ± 3.7	0.269 (0.05)	0.688 (0.01)	0.215 (0.06)	+ 0.03	(− 0.2, 0.2)	− 0.43	(− 0.4, 0.1)
Android/Gynoid mass ratio (g/g)	1.14 ± 0.24	1.13 ± 0.23	0.99 ± 0.15	1.00 ± 0.14	0.834 (0.00)	0.070 (0.13)	0.633 (0.01)	− 0.04	(− 0.05, 0.04)	+ 0.19	(− 0.02, 0.03)
Trunk/leg fat percentage ratio (%/%)	1.11 ± 0.23	1.12 ± 0.24	0.96 ± 0.17	0.98 ± 0.18	0.200 (0.07)	0.085 (0.11)	0.652 (0.01)	+ 0.16	(− 0.02, 0.03)	+ 0.35	(− 0.01, 0.04)
Trunk/leg fat mass ratio (g/g)	1.25 ± 0.33	1.29 ± 0.33	1.05 ± 0.20	1.13 ± 0.28	**0.021** (0.20)	0.126 (0.09)	0.450 (0.02)	+ 0.44	(− 0.02, 0.10)	+ 0.52	(− 0.01, 0.17)

Data are mean ± SD. BFR: blood flow restriction. Bolded values indicate *p* < 0.05. Effect columns show P (ηp², partial eta-squared: 0.01 small, 0.06 medium, 0.14 large). dz, within-group Cohen’s d for the pre-to-post change; 95% CI, 95% confidence interval of that change. Arm and leg lean mass are the mean of the left and right limb

**Table 2:** Blood markers of lipids and glucose metabolisms

Groups	BFR		Non-BFR		Time effect	Group effect	Interaction effect	BFR		Non-BFR	
	Pre	Post	Pre	Post				dz	95% CI	dz	95% CI
Total cholesterol (mmol/l)	6.0 ± 0.9	5.9 ± 0.7	5.4 ± 0.9	5.2 ± 1.0	0.344 (0.04)	**0.046** (0.15)	0.670 (0.01)	− 0.11	(− 0.42, 0.28)	− 0.25	(− 0.58, 0.23)
HDL cholesterol (mmol/l)	1.5 ± 0.2	1.4 ± 0.3	1.3 ± 0.3	1.2 ± 0.3	**0.008** (0.26)	0.147 (0.08)	0.251 (0.05)	− 0.28	(− 0.11, 0.04)	− 0.97	(− 0.13, − 0.03)
Total cholesterol/HDL ratio	4.2 ± 0.9	4.3 ± 0.8	4.3 ± 1.2	4.4 ± 1.3	0.518 (0.02)	0.797 (0.00)	0.610 (0.01)	+ 0.04	(− 0.23, 0.26)	+ 0.19	(− 0.27, 0.53)
Triglycerides (mmol/l)	1.0 ± 0.5	1.0 ± 0.5	1.3 ± 1.1	1.2 ± 0.9	0.624 (0.02)	0.473 (0.02)	0.123 (0.10)	+ 0.31	(− 0.070, 0.206)	− 0.34	(− 0.350, 0.090)
Fasting blood glucose (mmol/l)	5.5 ± 1.0	5.4 ± 0.9	4.7 ± 0.5	4.8 ± 0.4	0.910 (0.00)	**0.013** (0.22)	0.206 (0.06)	− 0.28	(− 0.35, 0.13)	+ 0.22	(− 0.15, 0.33)
HbA1c (NGSP/DCCT) (%)	5.4 ± 0.3	5.5 ± 0.3	5.3 ± 0.2	5.4 ± 0.3	**< 0.001** (0.46)	0.258 (0.05)	0.427 (0.03)	+ 0.56	(− 0.01, 0.21)	+ 1.68	(0.09, 0.19)
HbA1c (IFCC) (mmol/mol)	36.1 ± 3.6	37.2 ± 3.6	34.5 ± 2.8	36.0 ± 3.0	**< 0.001** (0.45)	0.277 (0.05)	0.463 (0.02)	+ 0.57	(− 0.1, 2.2)	+ 1.59	(1.0, 2.0)

Data are mean ± SD. BFR: blood flow restriction, HDL: high-density lipoproteins, HbA1c: glycated haemoglobin. Bolded values indicate *p* < 0.05. Effect columns show P (ηp², partial eta-squared: 0.01 small, 0.06 medium, 0.14 large). dz, within-group Cohen’s d for the pre-to-post change; 95% CI, 95% confidence interval of that change

**Table 3:** Strength of knee extensors and flexors

Groups	BFR		Non-BFR		Time effect	Group effect	Interaction effect	BFR		Non-BFR	
	Pre	Post	Pre	Post				dz	95% CI	dz	95% CI
Isometric peak torque (Nm)	186 ± 50	222 ± 80	183 ± 81	204 ± 90	**< 0.001** (0.40)	0.722 (0.01)	0.287 (0.05)	+ 0.78	(8, 65)	+ 0.95	(8, 34)
Isokinetic ext. at 30 °/s (Nm)	153 ± 36	166 ± 38	136 ± 60	155 ± 69	**0.003** (0.30)	0.485 (0.02)	0.546 (0.01)	+ 0.46	(− 4, 29)	+ 0.81	(5, 32)
Isokinetic flex. at 30 °/s (Nm)	109 ± 27	122 ± 29	100 ± 36	106 ± 34	**< 0.001** (0.38)	0.291 (0.04)	0.194 (0.07)	+ 0.79	(3, 23)	+ 0.79	(2, 11)
Isokinetic ext. at 90 °/s (Nm)	123 ± 51	158 ± 50	130 ± 61	140 ± 62	**0.032** (0.16)	0.789 (0.00)	0.213 (0.06)	+ 0.49	(− 8, 79)	+ 0.49	(− 2, 22)
Isokinetic flex. at 90 °/s (Nm)	74 ± 33	99 ± 29	76 ± 33	86 ± 30	**0.016** (0.21)	0.585 (0.01)	0.282 (0.05)	+ 0.51	(− 4.6, 54.0)	+ 0.82	(3.0, 17.0)
Isokinetic ext. at 180 °/s (Nm)	117 ± 41	127 ± 42	110 ± 50	116 ± 52	**0.022** (0.19)	0.627 (0.01)	0.606 (0.01)	+ 0.50	(− 2, 22)	+ 0.44	(− 2, 15)
Isokinetic flex. at 180 °/s (Nm)	74 ± 23	84 ± 25	65 ± 26	72 ± 25	**0.012** (0.22)	0.261 (0.05)	0.524 (0.02)	+ 0.50	(− 2.2, 22.7)	+ 0.66	(0.8, 11.8)

Data are mean ± SD. BFR: blood flow restriction, Ext.: extension, Flex.: flexion. Bolded values indicate *p* < 0.05. Effect columns show P (ηp², partial eta-squared: 0.01 small, 0.06 medium, 0.14 large). dz, within-group Cohen’s d for the pre-to-post change; 95% CI, 95% confidence interval of that change

**Table 4:** Gait energetics and biomechanics

Groups	BFR		Non-BFR		Time effect	Group effect	Interaction effect	BFR		Non-BFR	
	Pre	Post	Pre	Post				dz	95% CI	dz	95% CI
Wext (J kg^−1^ m^−1^)	0.26 ± 0.05	0.27 ± 0.05	0.26 ± 0.03	0.25 ± 0.03	0.760 (0.00)	0.623 (0.01)	0.188 (0.08)	+ 0.18	(− 0.014, 0.025)	− 0.43	(− 0.021, 0.004)
Recovery (%)	71.52 ± 5.23	71.31 ± 4.35	72.55 ± 4.61	72.49 ± 4.88	0.650 (0.01)	0.564 (0.01)	0.798 (0.00)	− 0.11	(− 1.4, 1.0)	− 0.07	(− 0.5, 0.4)
Net metabolic rate (W kg^−1^)	2.54 ± 0.36	2.63 ± 0.38	2.63 ± 0.53	2.48 ± 0.28	0.714 (0.01)	0.831 (0.00)	0.164 (0.08)	+ 0.24	(− 0.15, 0.34)	− 0.33	(− 0.45, 0.13)
Net cost of walking (J kg^−1^ m^−1^)	2.28 ± 0.32	2.37 ± 0.34	2.37 ± 0.48	2.23 ± 0.25	0.713 (0.01)	0.835 (0.00)	0.162 (0.08)	+ 0.24	(− 0.14, 0.30)	− 0.33	(− 0.40, 0.12)
Net locomotor efficiency	0.12 ± 0.03	0.11 ± 0.03	0.11 ± 0.05	0.11 ± 0.02	0.786 (0.00)	0.690 (0.01)	0.565 (0.01)	− 0.12	(− 0.013, 0.009)	+ 0.13	(− 0.022, 0.034)
Preferred walking speed (m s^−1^)	1.31 ± 0.14	1.33 ± 0.13	1.23 ± 0.24	1.27 ± 0.18	0.229 (0.06)	0.302 (0.05)	0.800 (0.00)	+ 0.19	(− 0.06, 0.12)	+ 0.31	(− 0.04, 0.12)

Data are mean ± SD. BFR: blood flow restriction, Wext, external mechanical work performed per step. Bolded values indicate *p* < 0.05. Effect columns show P (ηp², partial eta-squared: 0.01 small, 0.06 medium, 0.14 large). dz, within-group Cohen’s d for the pre-to-post change; 95% CI, 95% confidence interval of that change

Over the 6 weeks, knee-extensor and -flexor strength increased in both groups: in the BFR group the isometric and isokinetic torques rose by 8 to 33% and in the non-BFR group by 6 to 14%, with no group × time interaction for any strength outcome. Trunk lean mass increased by 2.8% (BFR) and 1.6% (non-BFR), whereas total, arm and leg lean mass did not change. HbA1c increased (by 2 to 3% across groups) and HDL-cholesterol decreased (by 2 to 6%), both small and directionally unfavourable shifts. Total body mass, BMI, total and regional fat, the remaining blood markers, and all walking energetic and biomechanical outcomes did not change significantly over time. No outcome showed a group × time interaction.

## Discussion

This study tested whether adding BFR to a 6-week, remotely delivered bodyweight resistance program would augment adaptations in body composition, metabolic health, strength, and walking function in sedentary middle-aged adults with overweight or obesity. Three findings stand out. First, knee flexor and extensor strength improved across all seven contractions tested, consistent with a meaningful neuromuscular response to bodyweight training in previously sedentary adults. Second, whole-body composition, gait energetics, and gait biomechanics were unchanged, and HDL-cholesterol, HbA1c, and the trunk-to-leg fat ratio shifted in directionally unfavourable ways small in magnitude and likely explained by the absence of dietary co-intervention. Third, and contrary to our hypothesis, adding BFR conferred no additional benefit for any outcome.

### Body composition

Total lean mass did not change with training, contrary to our hypothesis. A meta-analysis (Loenneke et al. 2012) shows that muscle hypertrophy can occur after four weeks of training with BFR and that these adaptations remain constant from four to ten weeks of training. Furthermore, two to three sessions per week are the training frequency for maximizing muscle mass gains along with isotonic exercise mode (as opposed to walking). In our study, the training duration (six weeks), frequency (three sessions/week), and mode (isotonic) correspond to these training modalities maximizing muscular hypertrophy. Despite this finding, hypertrophy is correlated with training duration and, although unclear, six to seven weeks of resistance training at reasonably high intensity are required to observe significant increases in fiber cross-sectional area (Phillips 2000). Therefore, the 6-week training duration in the present study, combined with exercises targeting multiple muscle groups instead of focusing on some body parts, may have resulted in insufficient training volume for each muscle, thus preventing any significant increase in lean mass.

A second consideration is that adherence and technical execution are harder to assure in home-based than in on-site programs (Argent et al. 2018) and more challenging to monitor than on-site programs. For instance, it is difficult to ensure a full range of motion and verify correct BFR pressure. This could explain the lack of observed differences in this parameter and other studied variables and reinforce the importance of personal trainers on site.

Despite no change in total lean mass, trunk lean mass increased with training. This result likely reflects the specific demands of the exercise protocol: push-ups, hip raises, and front lunges heavily engage the core and stabilizing musculature. This localized hypertrophy was likely sufficient to be detected within the trunk segment, even if the magnitude of change was not substantial enough to alter global lean mass metrics. Additionally, BFR typically exerts its effects at the limb level, but some evidence suggests that BFR systemic effects could impact muscles that are not directly beneath a BFR cuff (Dankel et al. 2016). This interpretation should be treated with caution: a single significant segmental change, in the absence of any change in total and without a group effect, may equally reflect DEXA segmental measurement variability.

Body mass, BMI, and total fat mass were unchanged in both groups, in contrast with meta-analytic evidence that BFR training can reduce anthropometric indices in individuals with overweight or obesity (Sun 2022). Similarly, most home-based physical activity interventions for overweight/obese adults of a systematic review (Power et al. 2022) reported reductions in weight or BMI. The absence of changes in some studies, including this one, may be attributed to the absence of dietary control, adherence issues previously mentioned and/or a potentially insufficient exercise intensity prescribed, resulting in insufficient stimulus to induce measurable adaptations.

### Glucose and fat metabolism

A small time effect was observed for HbA1c, with no group × time interaction. The direction of change is opposite to the reductions typically reported with exercise in general and overweight/obese populations (Jamka et al. 2022). Despite the theoretical rationale for enhanced glucose regulation with BFR (increased metabolic stress, fast-twitch fiber recruitment, and glucose transport pathways (Saatmann et al. 2021) the present findings do not support any additive benefit. Existing literature remains inconsistent: no effect of BFR during 6-month aerobic training (Tanaka and Takarada 2018), a decrease without control group (Satoh 2011), and no difference versus active control despite reductions (Su et al. 2024). Therefore, this study is the first to report a slight HbA1c increase with no group × time interaction, indicating that the change is not attributable to BFR. This increase is unlikely to be clinically meaningful and may be explained by the absence of dietary control, the short intervention duration relative to erythrocyte lifespan (~ 120 days), the lack of changes in body composition, and inter-individual variability amplified by small sample size. The group effect for fasting blood glucose (*P* = 0.013) reflects a baseline difference between the groups and, in the absence of any time or interaction effect, cannot be interpreted as a training-induced change.

Although a recent meta-analysis reported favourable changes in total cholesterol, triglycerides, and HDL with BFR training in overweight or obese adults (Sun 2022), none of these changes were observed in the present study. On the contrary, a small decrease with time was observed for HDL-cholesterol, which is generally considered detrimental. HDL levels are primarily influenced by factors such as physical activity, weight changes, smoking, alcohol consumption, and diet (Escolà-Gil et al. 2015). The most plausible explanation for these unexpected results is the lack of dietary control, as the other factors are unlikely to have changed significantly or in a manner that would lead to a decrease in HDL levels.

### Isokinetic strength

Every strength outcome improved over time, with no detectable between-group difference. In the absence of measurable changes in total or leg lean mass, these rapid gains are most plausibly attributable to neural adaptations—a pattern typical of the first 4–8 weeks of resistance training in untrained adults. A reciprocal pattern (hypertrophy preceding neural adaptation) has been proposed for low-load BFR (Loenneke et al. 2012) but is not confirmed by the present study.

When compared to the non-BFR intervention, BFR did not augment strength gains, contrary to earlier reports (Loenneke et al. 2012). While BFR superiority is often reported when performed with low load, it does not outperform traditional high-load resistance training (Perera et al. 2022). Thus, the low initial fitness level of the participants and the higher body-weight intensity of the present training program may have overshadowed any BFR advantage at lower intensities. These results align with previous findings from our research group in elderly individuals (Citherlet et al. 2025): exercise specificity appears to be less critical in unfit participants, particularly during the early stages of a training intervention.

### Gait energetics and biomechanics

Walking energetics and biomechanics did not change with training in either group, with no evidence of a BFR-specific effect. In contrast, a meta-analysis (Hortobágyi et al. 2015) demonstrated that resistance training can increase preferred walking speed in older adults by 0.10 ± 0.12 m/s. Several factors may explain this difference: (1) most studies included in the meta-analysis reported no significant effect, suggesting that the pooled significant effect may result from the increased statistical power of the meta-analysis. Thus, small-scale studies, such as ours, may lack statistical power to detect similar changes; (2) older adults are more likely to experience these improvements because resistance training counteracts age-related muscle loss and enhances neuromuscular function, which are key factors in walking speed (Cruz-Jentoft et al. 2019). In contrast, obese adults’ walking speed may be more influenced by excess body weight, which did not change in the present study; (3) the baseline preferred walking speed in our study (1.27 m s⁻¹) was faster than that reported for individuals with obesity (≈ 1.07 m s⁻¹; (Malatesta et al. 2022), more similar to that of normal-weight individuals (≈ 1.34 m s⁻¹; (Majed et al. 2022). This indicates that our resistance training may find it harder to improve walking speeds that are already relatively high; (4) some studies were better designed to improve walking speed specifically. For example, one study (Uematsu et al. 2014) had the participants required to perform resistance training at high velocity to meet the specificity of the training stimulus required to improve walking speed, which was not the case in our study, where participants had a slow pace of execution.

Individuals with obesity display a higher relative net cost of walking than their lean counterparts due to a less efficient gait pattern that necessitates a greater volume of muscle work, leading to reduced mechanical efficiency (Fernández Menéndez et al. 2019), although the magnitude of this difference is not uniform across obesity classes (Primavesi et al. 2021; Tucker et al. 2024). However, they have a lower relative Wext along with higher levels of recovery because of a better inverted-pendular mechanism, thus limiting the increase in the relative net cost of walking (Fernández Menéndez et al. 2020). In the present study, the participant did, nevertheless, not show any improvements regarding Wext or the net cost of walking, resulting in no significant changes in net locomotor efficiency. In comparison, large (Malatesta et al. 2022) but not small (Peyrot et al. 2010) weight loss increased Wext and decreased recovery, while the net cost of walking decreased in both studies. The absence of body weight change in the present study likely explains the absence of improvements in Wext, recovery, net cost of walking, or net locomotor efficiency.

BFR did not lead to any difference although the potential peripheral adaptations following BFR resistance training (improved muscle oxygen delivery and improved mitochondrial respiratory function) could have lowered the net cost of walking. Nevertheless, our results align with previous reports showing that low-load resistance training increases strength, with no further improvement with the application of BFR (Pignanelli et al. 2020).

### Methodological considerations

Several limitations warrant consideration. First, the absence of dietary intervention meant participants’ nutritional habits were not optimized for body composition changes. Second, the home-based nature of the study, while enhancing ecological validity, complicated the oversight of exercise technique, range of motion, and BFR pressure. Although objective adherence was monitored via session attendance and self-reports, the lack of direct observation outside of Zoom sessions may have influenced protocol fidelity. Third, this study used fixed cuff pressures, reflecting the real-world conditions of remotely supervised BFR training, where participants would not typically measure arterial occlusion pressure (AOP). Although these pressures have previously been shown to restrict blood flow in healthy individuals (Citherlet et al. 2022), AOP was not measured individually, likely resulting in a heterogeneous physiological stimulus. Because AOP varies with factors such as limb circumference, subcutaneous fat thickness, and blood pressure, the relative level of blood flow restriction likely differed between participants, particularly those with higher adiposity who may require greater cuff pressures to achieve comparable restriction (Karabulut and Garcia 2017). Fourth, the baseline sex distribution differed between groups (38% vs. 71% female), potentially confounding between-group comparisons. A sensitivity analysis adding sex as a covariate to the group × time comparisons did not render any interaction significant, so the between-group findings are robust to this imbalance. Fifth, the modest sample size (*n* = 27) also limited the statistical power to detect small-to-moderate effects. Consequently, the lack of group × time interactions should be interpreted as a lack of evidence for superiority rather than proof of equivalence. Finally, the 6-week duration, while adequate for detecting early neuromuscular adaptations, is short relative to the time-course required for measurable changes in body composition (typically  ≥ 12 weeks) and HbA1c (which reflects glycaemia over the preceding  ~ 120 days).

## Conclusion

In conclusion, the 6-week remote resistance training program significantly improved lower-limb muscle strength in sedentary individuals with overweight and obesity, but had no beneficial effect on body composition, glucose and lipid markers, or gait energetics and biomechanics; HDL-cholesterol and HbA1c showed small, statistically significant but clinically negligible shifts in an unfavourable direction, most plausibly reflecting the absence of dietary control. There was no evidence that BFR provides superior benefits compared to non-BFR resistance training. These findings underscore the potential of home-based resistance training to improve key health markers but suggest that BFR may not be advantageous in this context. Future studies should compare the effects of remote resistance training with other types of exercise, such as aerobic or combined resistance and aerobic training, to assess whether the lack of improvement in body composition and gait energetics is specific to resistance training or common across different modalities.

### Practical applications

For strength and conditioning practitioners working with sedentary adults with overweight or obesity, the present findings support three practical recommendations. First, a low-cost, home-based bodyweight resistance training program delivered via supervised video-conferencing (three 30–35-min sessions week⁻¹ for 6 weeks) produced meaningful improvements in knee extensor and flexor strength in both BFR and non-BFR groups, making it a viable entry-level option for individuals who are unable or unwilling to attend a gym. Second, the addition of pneumatic BFR cuffs at the prescribed absolute pressures did not confer additional benefits for strength, body composition, blood lipid/glucose metabolism, or walking energetics; practitioners should therefore weigh the incremental cost, time to apply/monitor cuffs, and participant tolerance against the modest added value demonstrated here. If BFR is employed with this population, practitioners should personalise cuff pressure to a target %AOP measured individually (e.g., 40–80% AOP using a Doppler-calibrated device) rather than using fixed absolute pressures, because effective occlusion varies with individual characteristics. Third, remote delivery requires that clinicians pair resistance training with explicit nutritional guidance and objective adherence tracking; without dietary control, the 6-week program was insufficient to change body mass, BMI, or total fat mass, and therefore should not be recommended as a stand-alone weight-loss strategy. Longer interventions (≥ 12 weeks) combining resistance training with structured nutrition counselling are likely required to shift body composition in this population.

## Data Availability

Data are available from the corresponding author upon request.

## Abbreviations

BFR: Blood flow restriction
BMI: Body mass index
HbA1c: Glycated haemoglobin
HDL: High-density lipoproteins
${\dot{\text {VCO}}_{2}}$: Carbon dioxide production
$${\dot{\text {VO}}_{2}}$$: Oxygen uptake
